# Physics-based reconstruction methods for magnetic resonance imaging

**DOI:** 10.1098/rsta.2020.0196

**Published:** 2021-06-28

**Authors:** Xiaoqing Wang, Zhengguo Tan, Nick Scholand, Volkert Roeloffs, Martin Uecker

**Affiliations:** ^1^ Institute for Diagnostic and Interventional Radiology, University Medical Center Göttingen, Göttingen, Germany; ^2^ Partner Site Göttingen, German Centre for Cardiovascular Research (DZHK), Göttingen, Germany; ^3^ Cluster of Excellence ‘Multiscale Bioimaging: from Molecular Machines to Networks of Excitable Cells’ (MBExC), University of Göttingen, Göttingen, Germany; ^4^ Campus Institute Data Science (CIDAS), University of Göttingen, Göttingen, Germany

**Keywords:** magnetic resonance imaging, model-based reconstruction, inverse problems

## Abstract

Conventional magnetic resonance imaging (MRI) is hampered by long scan times and only qualitative image contrasts that prohibit a direct comparison between different systems. To address these limitations, model-based reconstructions explicitly model the physical laws that govern the MRI signal generation. By formulating image reconstruction as an inverse problem, quantitative maps of the underlying physical parameters can then be extracted directly from efficiently acquired k-space signals without intermediate image reconstruction—addressing both shortcomings of conventional MRI at the same time. This review will discuss basic concepts of model-based reconstructions and report on our experience in developing several model-based methods over the last decade using selected examples that are provided complete with data and code.

This article is part of the theme issue ‘Synergistic tomographic image reconstruction: part 1’.

## 1. Introduction

The first physics-based reconstruction methods for parametric mapping appeared in the literature more than a decade ago [1–3] and constitute now a major research area in the field of magnetic resonance imaging (MRI) [4–13]. Model-based reconstruction is based on modelling the physics of the MRI signal and has been used, for example, to estimate *T*_1_ [6,9,13–16], *T*_2_ relaxation [3,5,10,17], for $T_{2}^{⋆}$ estimation and water-fat separation [18–22], as well as for quantification of flow [11] and diffusion [23,24]. Quantitative maps of the underlying physical parameters can then be extracted directly from the measurement data without intermediate image reconstruction. This direct reconstruction has two major advantages: first, the full signal is described by a model based on a few parameter maps only and intermediate image reconstruction is waived. This renders model-based techniques much more efficient in exploiting the available information than conventional two-step methods. Second, as a specific signal behaviour is no longer required for image reconstruction, MRI sequences can now be designed that have an optimal sensitivity to the parameters of interest. Once the underlying physical parameters are estimated, arbitrary contrast-weighted images can be generated synthetically by evaluating the signal model for a specific sequence and acquisition parameters.

In this work, we discuss our experience using model-based reconstruction methods with different radial MRI sequences showing a variety of examples ranging from *T*_1_ and *T*_2_ mapping and banding-free bSSFP imaging in the brain over flow quantification in the aorta to water-fat separation and $T_{2}^{⋆}$ mapping in the liver. All provided examples come with data and code and can be reconstructed using the BART toolbox [25].

## 2. MRI signal

In typical MRI experiments, the proton spins are polarized by bringing them into a strong external field. The spins then start to precess with a characteristic Larmor frequency and can be manipulated using additional on-resonant radio-frequency pulses and further gradient fields. The dynamical behaviour of the magnetization is described by the Bloch–Torrey equations that describe the physics of magnetic resonance including effects from relaxation, flow and diffusion. As a fully computer-controlled imaging method, MRI is extremely flexible and the underlying physics enables access to a variety of tissue and imaging system-specific parameters such as relaxation constants, flow velocities, diffusion, temperature, magnetic fields, etc.

The measured MRI signal corresponds to the complex-valued transversal magnetization *M* which is obtained by quadrature demodulation from the voltage induced in the receive coils. In a multi-coil experiment, this signal is proportional to the transversal magnetization weighted by the sensitivity of each receive coil:

2.1 $$y_{j}(t)=\int c_{j}(r)\;M(x,B,t,r)\;dr.$$

Here, *c*_*j*_ is the complex-valued sensitivity of the *j*th coil and *M* the complex-valued transversal magnetization at time *t* and position ***r***. The magnetization depends on some physical parameters *x* and the externally controlled magnetic fields *B*(*t*, ***r***), i.e. gradient fields and radio-frequency pulses, and can be obtained by solving the Bloch–Torrey equations (or, if motion of spins can be neglected, by solving the Bloch equations at each point).

While equation (2.1) can be exploited directly for model-based reconstruction [26], many other model-based methods use some simplifying approximations to reduce the computational complexity. Often, segmentation in time is used by assuming that the magnetization is constant around certain time points, e.g. around echo times TE_*n*_ with n∈1,…,N. *N* is the number of echos. The effect of magnetic field gradients can then be separated out into a phase term which is defined by the k-space trajectory ***k***(*t*). This separation often allows the use of simplified models for the magnetization and, more importantly, the use of fast (non-uniform) Fourier transform algorithms for the gradient-encoding term. We derive the following—still very generic—model:

2.2 $$y_{n,j}(t)=\int e^{i2\pi r\cdot k(t)}c_{j}(r)M(x,t_{n},r)\;dr.$$

**Figure 1.**
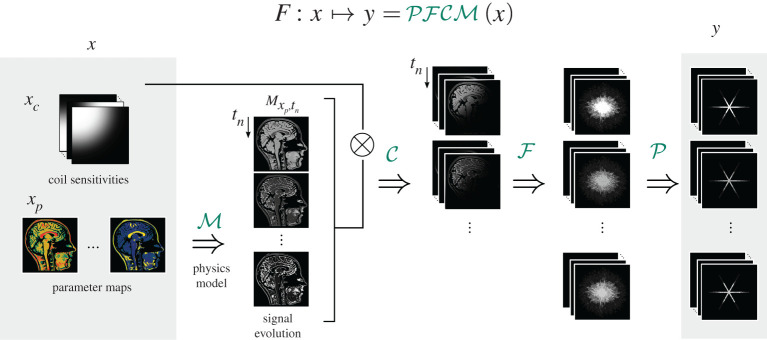
The forward operator *F* can be formally factorized into operator M that describes the spin physics, the multiplication with the coil sensitivities C, the (non-uniform) Fourier transform F, and a sampling operator P. (Online version in colour.)

Based on this model, we define a nonlinear forward operator F:x↦y that maps the unknown parameters *x* to the acquired data *y*. This operator can be formally decomposed into F=PFCM, where P is the sampling operator, F is the Fourier transform, C the multiplication with the coil sensitivities, and M the signal model (figure 1).

The defining feature of physics-modelling reconstruction is the addition of a signal model M into the forward operator *F*. The specific signal model depends on the applied sequence protocol and specifies which tissue and/or hardware characteristics can be estimated. Often, an analytical model can be derived from the Bloch equations using hard-pulse approximations. For many typical MRI sequences important parameter dependencies are exponentials. Table 1 lists some of these analytical signals models. If the applied sequence protocol does not lend itself to an analytical signal expression, the Bloch equations need to be integrated as signal model directly. This integration becomes challenging for iterative reconstructions, because of the estimation of the signals derivatives. Current techniques exploit finite difference methods [10,26] or sensitivity analysis of the Bloch equations [27].

**Table 1.** Basic analytical signal models for physical parameter dependencies in common MRI sequences.

parameter	sequence type	signal
relaxation rate *R*_1_	inversion recovery	$a-(1+a)\cdot e^{-t_{n}R_{1}/a}$
relaxation rate *R*_2_	spin-echo	$e^{-R_{2}t_{n}}$
relaxation rate $R_{2}^{⋆}$	gradient-echo	$e^{-R_{2}^{*}t_{n}}$
field *B*_0_	gradient-echo	$e^{i2\pi \cdot f_{B_{0}}t_{n}}$
chemical shift	gradient-echo	$\sum_{p}a_{p}{\text{e}}^{\text{i2}\pi f_{p}t_{n}}$
flow velocity ***v***	bipolar gradient	$e^{iv\cdot V_{n}}$
diffusion tensor *D*	bipolar gradient	$e^{-b_{n}^{T}Db_{n}}$

The MRI data used in this work was acquired on a Siemens Skyra 3T scanner (Siemens Healthcare GmbH, Erlangen, Germany) from four volunteers (21–35 years, two females) without known illness after obtaining written informed consent and with approval of the local ethics committee. Acquisition parameters can be found in table 2.

**Table 2.** Detailed parameters of MR sequences capable of mapping physical parameters listed in table 1.

sequence	flip angle °	TR/TE/ΔTE ms	bandwidth Hz/px	matrix	spokes	TA s	FOV mm	slice mm
IR-FLASH	6	4.10/2.58	630	256 × 256	1020	4	192	5
ME-SE	90/180	2500/9.9/9.9	390	256 × 256	25 × 16	80	192	3
ME-FLASH	5	10.60/1.37/1.34	960	200 × 200	33 × 7	0.35^*a*^	320	5
PC-FLASH	10	4.46/2.96	1250	210 × 210	2 × 7	15	320	5
fmSSFP^*b*^	15	4.5/2.25	840	192 × 192	4 × 101 × 40	137	192	1

^*a*^Acquisition time per frame, because the presented example is based on dynamic acquisition. ^*b*^3D Stack-Of-Stars sequence with 40 partitions (1000 prep scans), while all other acquisition protocols are 2D.

## 3. Nonlinear reconstruction

Using a nonlinear forward operator F:x↦y that maps the unknown parameters *x* to the acquired data *y*, we can formulate the image reconstruction as a nonlinear optimization problem:

3.1 $$\hat{x}=\underset{x}{\mathrm{argmin}}||F(x)-y|{|}_{2}^{2}+\sum_{i}\lambda_{i}R_{i}(x).$$

Data fidelity is ensured by $||F(x)-y|{|}_{2}^{2}$ and regularization terms *R*_*i*_ can be added to introduce prior knowledge with *λ*_*i*_ the corresponding regularization parameters. This framework is very general, combining parallel imaging, compressed sensing (CS), and model-based reconstruction in a unified reconstruction. Often the coil sensitivities are estimated before, but they could also be included as unknowns in *x*. Paired with suitable sampling schemes, this yields fully calibrationless methods that do not require additional calibration scans [11,13,28–31]. Moreover, model-based reconstructions allow a direct application of sparsity-promoting regularizations to the physical parameters for performance improvement [3,7,13,24].

However, the high non-convexity of model-based reconstruction makes this method sensible to the initial guess and relative scaling of the derivatives of each parameter map. These issues can often be addressed with a reasonable initial guess and a proper preconditioning. Algorithms to solve the nonlinear inverse problems include gradient descent, the variable projection methods [7,32], the method of nonlinear conjugate gradient [33] and Newton-type methods [34]. Particularly for the examples presented in this paper, we solve equation (3.1) via an iteratively regularized Gauss–Newton method (IRGNM) [34] where the nonlinear problem in equation (3.1) is linearized in each Gauss–Newton step, i.e.

3.2 $${\hat{x}}_{n+1}=\underset{x}{\mathrm{argmin}}||DF(x_{n})(x-x_{n})+F(x_{n})-y|{|}_{2}^{2}+\sum_{i}\lambda_{i}R_{i}(x),$$

with *DF*(*x*_*n*_) the Jacobian matrix of *F* at the point *x*_*n*_. The regularized linear subproblem can be further solved by conjugate gradients, FISTA [35] or ADMM [36].

### (a) *T*_1_ and *T*_2_ mapping

*T*_1_ mapping can be accomplished using a inversion-recovery (IR) FLASH sequence: following a inversion pulse, data are continuously acquired using the FLASH readout. The magnetization signal *M*(*t*) for IR-FLASH reads [37,38]

3.3 $$M_{t_{k}}(r)=M_{\mathrm{ss}}(r)-\left(M_{\mathrm{ss}}(r)+M_{0}(r)\right)\cdot e^{-t_{k}\cdot R_{1}^{*}(r)},$$

with *M*_ss_ the steady-state magnetization, *M*_0_ the equilibrium magnetization, and R*_1_ the effective relaxation rate, i.e. R*_1_ = 1/T*_1_. *t*_*k*_ is the inversion time defined as the centre of each acquisition window. The acquisition window is determined by the product of repetition time and the number of readouts formulating one k-space (binning) after inversion. Although model-based reconstructions do not require any binning, this process is helpful for reducing the computation demand while still keeping the *T*_1_ accuracy [13]. After estimation of *M*_ss_, *M*_0_ and R*_1_, *T*_1_ then can be calculated afterwards by: *T*_1_ = *M*_0_/(*M*_ss_ · R*_1_). Data for *T*_1_ mapping are acquired using a single-shot IR radial FLASH (4 s) sequence with a tiny golden angle (≈23.36°) between successive spokes.

The multi-echo spin echo (ME-SE) sequence can be employed for *T*_2_ mapping. The magnetization signal *M*(*t*) for a multi-echo spin echo sequence at echo time *t*_*k*_ follows an exponential decay $M_{t_{k}}(r)=M_{0}^{\prime }\cdot e^{-t_{k}\cdot R_{2}(r)}$ with *M*′_0_ the spin density map, *R*_2_ = 1/*T*_2_ the transverse relaxation rate. This simple exponential model does not take stimulated echoes into account, but a more complicated analytical model exists for this case [39,40]. Data for *T*_2_ mapping are obtained with 25 excitations and 16 echoes per excitation using a radial golden-ratio (≈111.25°) sampling strategy.

Quantitative parameter maps for both acquisitions are estimated using the nonlinear model-based reconstruction. In other words, the estimation of parameter maps (*M*_ss_, *M*_0_, R*_1_)^*T*^ or parameter maps (*M*′_0_, *R*_2_)^*T*^, respectively, and coil sensitivity maps (*c*_1_, …, *c*_*N*_)^*T*^ is formulated as a nonlinear inverse problem with a joint ℓ_1_-Wavelet regularization applied to the parameter maps and the Sobolev norm [41] to the coil sensitivity maps. This nonlinear inverse problem is then solved by the IRGNM-FISTA algorithm [13]. After estimation of the parameters *T*_1_ and *T*_2_ maps can be calculated. Note that the *M*_0_ and *M*′_0_ absorb physical effects which are not explicitly modelled and identical over all inversion or echo times.

To evaluate the quantitative accuracy of the model-based methods, numerical phantoms with different *T*_1_ relaxation times (ranging from 200 ms to 2000 ms with a step size of 200 ms for each tube, and 3000 ms for the background), *T*_2_ relaxation times (ranging from 20 ms to 200 ms with a step size of 20 ms for each tube, and 1000 ms for the background) were simulated, respectively. To avoid an inverse crime [42], the *k*-space data was derived from the analytical Fourier representation of an ellipse assuming an array of eight circular receiver coils surrounding the phantom without overlap. Complex white Gaussian noise with a moderate standard deviation was added to the simulated *k*-space data.

**Figure 2.**
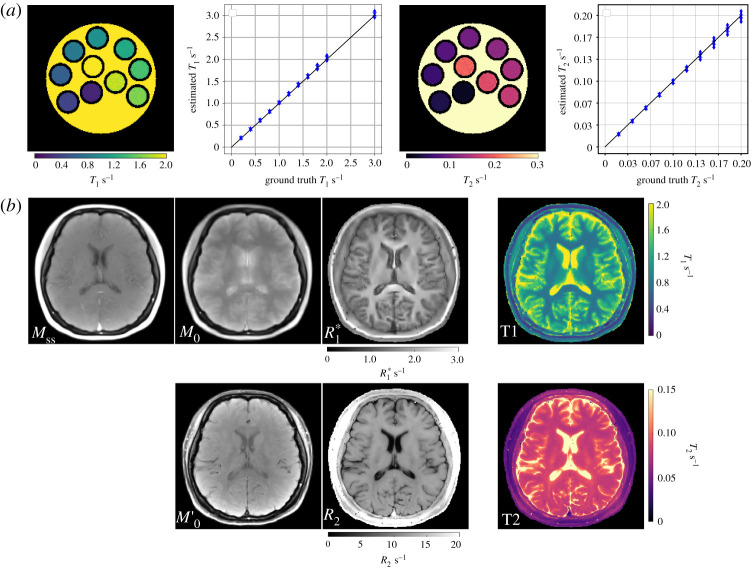
(*a*) (Leftmost) Model-based reconstructed *T*_1_ map and (left middle) the ROI-analysed quantitative *T*_1_ values for the numerical phantom using the single-shot IR radial FLASH sequence. (Right middle and rightmost) Similar results for *T*_2_ mapping using the multi-echo spin-echo sequence. (*b*) (Top) The reconstructed parameter maps (*M*_ss_, *M*_0_, R*_1_)^*T*^ for the *T*_1_ model and (bottom) (*M*_0_, *R*_2_)^*T*^ for the *T*_2_ model with the corresponding *T*_1_ / *T*_2_ maps in the rightmost column. (Online version in colour.)

Figure 2*a* presents the estimated *T*_1_, *T*_2_ maps and the corresponding ROI-analysed quantitative values for the numerical phantom using model-based reconstructions. Good quantitative accuracy is confirmed for both model-based *T*_1_ and *T*_2_ mapping methods. Figure 2*b* demonstrates model-based reconstructed three and two physical parameter maps, the corresponding *T*_1_ and *T*_2_ maps for the retrospective *T*_1_ and *T*_2_ models on human brain studies. Furthermore, synthetic images were computed for all inversion/echo times and the image series was then converted into movies showing the contrast changes in electronic supplementary material, videos 1 and 2. For the data presented here, model-based *T*_1_ and *T*_2_ reconstruction took around 6 and 3 min on a GPU (Tesla V100 SXM2, NVIDIA, Santa Clara, CA), respectively.

### (b) Water/fat separation and $R_{2}^{*}$ mapping

Quantitative $T_{2}^{*}$ mapping can be achieved via multi-echo gradient-echo sampling. With prolonged echo-train readout, the acquired multi-echo signal is

3.4 $$M_{n}=\rho \cdot e^{-R_{2}^{*}\cdot {\mathrm{TE}}_{n}}\cdot e^{i2\pi \cdot f_{B_{0}}\cdot {\mathrm{TE}}_{n}},$$

where $R_{2}^{*}$ is the inverse of $T_{2}^{*}$ and $f_{B_{0}}$ is the *B*_0_ field inhomogeneity. TE_*n*_ denotes the *n*th echo time. On the other hand, when the imaging voxel contains distinct protons resonating at different frequencies, the magnetization *ρ* can be split into multiple compartments. For instance, chemical shift between water and fat induces phase modulation, therefore,

3.5 $$M_{n}=(W+F\cdot \sum_{p}a_{p}e^{i2\pi f_{p}\cdot {\mathrm{TE}}_{n}})\cdot e^{-R_{2}^{*}\cdot {\mathrm{TE}}_{n}}\cdot e^{i2\pi \cdot f_{B_{0}}\cdot {\mathrm{TE}}_{n}},$$

where W and F are the water and fat magnetization, respectively. *f*_*p*_ is the *p*th fat-spectrum peak frequency, and *a*_*p*_ is the corresponding amplitude. In practice, usually the 6-peak fat spectrum [43] is used. In the model-based reconstruction formulation, the unknowns contain W, F, $R_{2}^{*}$ and $f_{B_{0}}$, as well as a set of coil sensitivity maps from the parallel imaging model.

**Figure 3.**
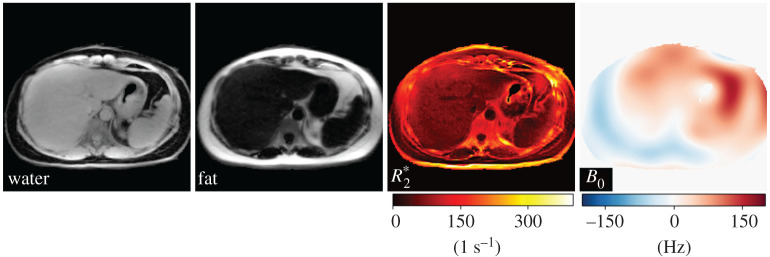
Real-time liver images acquired during free-breathing using a radial multi-echo (ME) FLASH sequence. Model-based reconstruction directly and jointly estimates separated water and fat images, as well as $R_{2}^{⋆}$ and *B*_0_ field maps. (Online version in colour.)

Here, a multi-echo (ME) radial FLASH sequence [31] was used to acquire liver data during free breathing. The model-based reconstruction was initialized by the estimate from model-based 3-point water/fat separation [31], while $R_{2}^{*}$ and coil sensitivity maps were initialized with 0. Afterwards, joint estimation of all unknowns in equation (3.5) including coil sensitivity maps was achieved via IRGNM with ADMM. The Sobolev-norm weight [41] was applied to the *B*_0_ field inhomogeneity and coil sensitivity maps. Joint ℓ1-Wavelet regularization was applied to other parameter maps. As shown in figure 3 and electronic supplementary material, video 3, high-quality respiratory-resolved water/fat separation as well as $R_{2}^{*}$ and $f_{B_{0}}$ maps can be achieved even with undersampled multi-echo radial acquisition (33 spokes per echo and 7 echoes in total).

### (c) Phase-contrast velocity mapping

In phase-contrast flow MRI, velocity-encoding gradients are used to encode flow-induced phases. Due to the complexity of MR signal, a reference measurement without flow-encoding gradients is required such that the phase difference between these two measurements excludes the background phase. Therefore, the phase-contrast flow MRI signal can be modelled as

3.6 $$M_{k}=\rho \cdot e^{iv\cdot V_{k}}\;.$$

***v*** is the velocity and *V*_*k*_ is the velocity-encoding for the *k*th measurement. For through-plane velocity mapping, *V*_0_ = 0 for the reference and *V*_1_ = *π*/VENC the velocity-encoded measurement, respectively. VENC is the maximum measurable velocity. *ρ* is the shared anatomical image between the two measurements.

**Figure 4.**
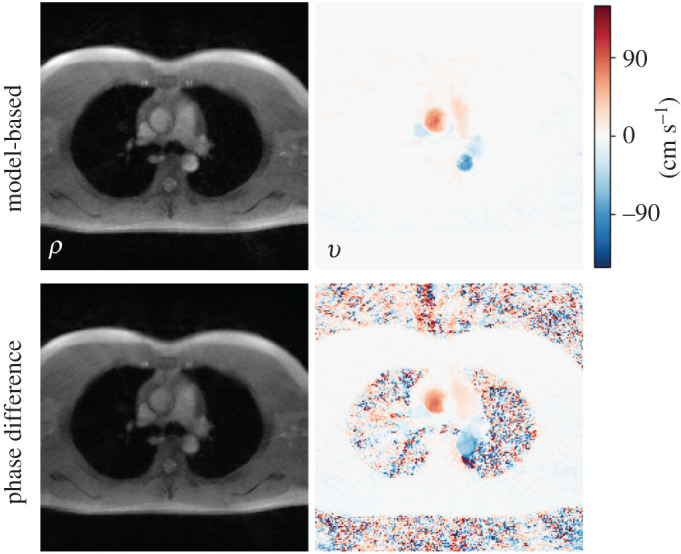
Comparison between (top) the model-based reconstruction and (bottom) the conventional phase-difference reconstruction. A section crossing the ascending and descending aorta was selected as the imaging slice. Displayed images are (left) anatomical magnitude image and (right) phase-contrast velocity map at systole. With direct phase-difference regularization, the model-based reconstruction largely reduces random background phase noise in the velocity map. (Online version in colour.)

As an example, flow MRI sequence with radial sampling and through-plane velocity-encoding gradient was used to measure aortic blood flow velocities. As shown in figure 4, with direct regularization on the phase-difference map, the proposed model-based reconstruction [11,30] is able to largely remove background random phase noise. Electronic supplementary material, video 4 displays the dynamic velocity maps of the whole 15 s scan.

## 4. Linear subspace reconstruction

In contrast to nonlinear models, in linear subspace methods the signal curves t↦M(x,t,r) are approximated using a linear combination of basis functions [44–52], i.e.

4.1 $$M(x,t,r)\approx \sum_{s}a_{s}(r)B_{s}(t)\;.$$

The linear basis functions *B*_*s*_(*t*) can be generated by simulating a set of representative signal curves for a range of parameters and performing a singular value decomposition to obtain a good representation.

With known coil sensitivities, this leads to a linear inverse problem for the subspace coefficients:

4.2 $$\hat{a}=\underset{a}{\mathrm{argmin}}||PFCBa-y|{|}_{2}^{2}+\sum_{i}\lambda_{i}R_{i}(a).$$

After reconstruction of the subspace coefficients *a*_*s*_, the parameters *x* need to be estimated in a separate step. This can be achieved by predicting complete magnetization maps for all time points and fitting a nonlinear signal model. This can be done point-wise, so is much easier than doing a full nonlinear reconstruction. Still, for multi-parametric mapping efficient techniques to map between coefficients and parameters are required [51].

Linear subspace methods have several advantages. Linear subspace models lead to linear inverse problems which do not have local minima. Due to their linearity, they also inherently avoid model violations stemming from partial volume effects. Because the matrix multiplication with the basis commutes with other operations that are identical at each time point, it is possible to combine the basis with the sampling operator. The reconstruction then admits a computationally advantageous formulation that allows computation to be performed entirely in the subspace [46,47].

### (a) *T*_1_ Mapping

**Figure 5.**
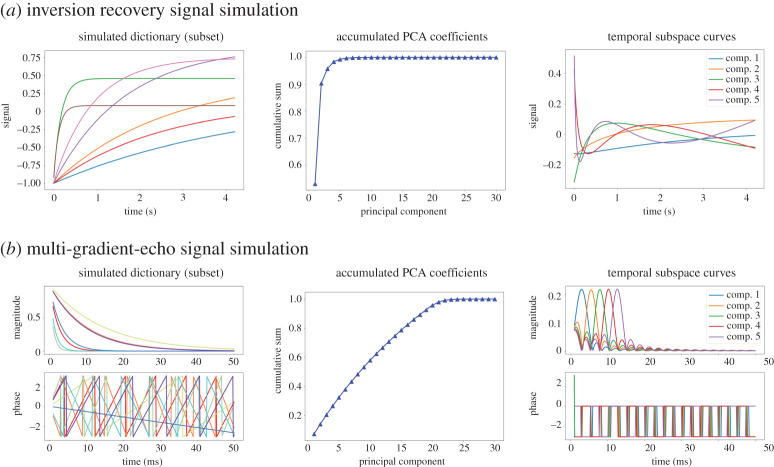
Demonstration of subspace-based methods for (*a*) single-shot inversion-recovery and (*b*) multi-gradient-echo signal, respectively. (Left) Simulated (top) *T*_1_ relaxation and (bottom) $T_{2}^{*}$ relaxation and off-resonance phase modulation curves. (Centre) Plot of the first 30 principle components. (Right) The temporal subspace curves that can be linearly combined to form (top) *T*_1_ relaxations and (bottom) multi-gradient-echo relaxations. (Online version in colour.)

Alternatively, *T*_1_ maps were also reconstructed using the subspace method. Similar to [50], the *T*_1_ dictionary was constructed using 1000 different 1/R*_1_ values linearly ranging from 5 to 5000 ms, combined with 100 *M*_ss_ values from 0.01 · *M*_0_ to *M*_0_. This results in 100 000 exponential curves in the dictionary. A subset of such a dictionary is shown in figure 5 (left). The other parameters are TR = 4.10 ms, 20 spokes per frame, 51 frames in total. The simulated curves are highly correlated and can be represented by only a few principle components (figure 5). For easier comparison, the subspace-constraint reconstruction used the coil sensitivity maps estimated using model-based *T*_1_ reconstruction. The resulting linear problem was then solved using conjugate gradient or FISTA algorithm in BART. The coefficient maps were then projected back to image series where the 3-parameter fit is applied for each voxel according to equation (3.3).

**Figure 6.**
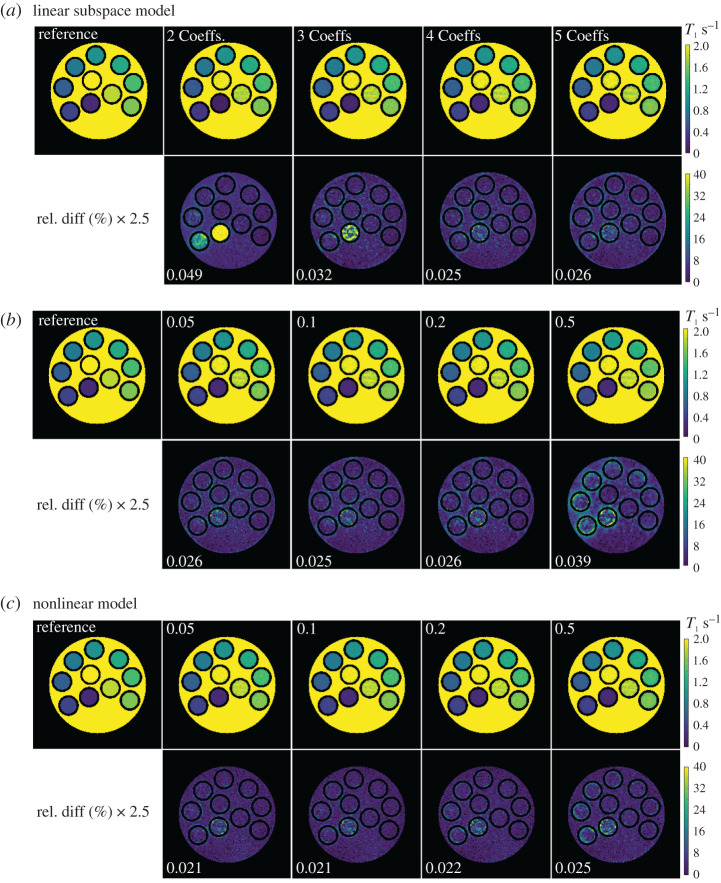
Comparison of linear and nonlinear model-based reconstructions on the simulated phantom. (*a*) Linear subspace reconstructed *T*_1_ maps using 2, 3, 4, 5 complex coefficients and their relative difference to the reference. (*b*) Linear subspace reconstructed *T*_1_ maps using four complex coefficients with changing regularization parameters. (*c*) Model-based reconstructed *T*_1_ maps using different regularization strengths. The numerical phantom used here is simulated using 208 frames, one spoke per frame, and TR = 20.5 ms. All reconstructions are done with L2-regularization. The regularization strength and the normalized relative errors to the reference are shown on the top-left and bottom-left of each figure, respectively. (Online version in colour.)

Figure 6*a* shows estimated phantom *T*_1_ maps using a variant number of complex coefficients of the linear subspace-based reconstruction with L2 regularization. A lower number of coefficients causes bias for quantitative *T*_1_ mapping (especially for tubes with short *T*_1_s) while a higher number of coefficients brings noise in the final *T*_1_ maps. Therefore, four coefficient maps were chosen to compromise between quantitative accuracy and precision. Figure 6*b* compares the effects of regularization strength. Similarly, low value of the regularization parameter brings noise while high regularization strength causes bias. A value of 0.1 was then chosen to compromise *T*_1_ accuracy and precision. Figure 6*c* then shows the effects of regularization for the model-based reconstruction. A value of 0.1 was selected as it has the least normalized error.

The low normalized relative errors on the optimized *T*_1_ maps reflect that both linear subspace and nonlinear model-based methods can generate *T*_1_ maps with good accuracy while nonlinear model-based reconstruction has a slightly better performance (i.e. less normalized relative errors).

**Figure 7.**
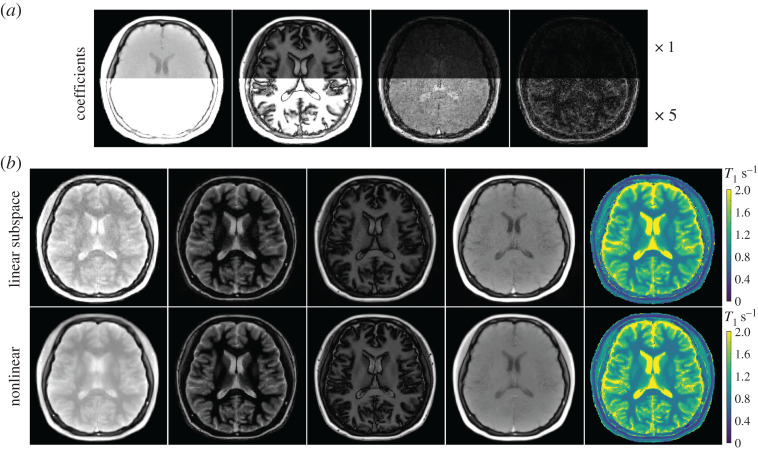
(*a*) Reconstructed four complex coefficient maps (only magnitude is shown) using the linear subspace method for a human brain study. (*b*) Synthesized images (at inversion time 40 ms, 400 ms, 800 ms, 4000 ms) using (top) the above four complex coefficient maps of the linear subspace method and (bottom) the three physical maps of the nonlinear model-based reconstruction, respectively. The corresponding *T*_1_ maps are presented in the rightmost column. (Online version in colour.)

With the above settings, figure 7*a* depicts the four main coefficient maps estimated using the linear subspace method for a brain study. In this case, a joint ℓ_1_-Wavelet sparsity regularization was applied to the maps with a strength of 0.0015 to improve the precision. For this dataset, the reconstruction together with a pixel-wise fitting took around 2 min on the GPU. Figure 7*b* presents the synthesized images along with the corresponding *T*_1_ maps using (top) the above four coefficient maps for the linear subspace and (bottom) the three physical parameter maps for nonlinear model-based reconstructions, where a similar joint ℓ_1_-Wavelet sparsity is applied with the regularization parameter 0.09. Again, both linear subspace and nonlinear methods could generate high-quality synthesized images and *T*_1_ maps while the nonlinear methods have slightly less noise and better sharpness.

Although linear subspace reconstruction has been demonstrated to be a fast and robust quantitative parameter mapping technique, it might not be directly applicable to MR signals with phase modulation along echo trains. For instance, multi-gradient-echo signals are known to be modulated by off-resonance-induced phases. A dictionary of multi-gradient-echo magnitude and phase signals was simulated with 256 × 256 $T_{2}^{*}$ and $f_{B_{0}}$ combinations linearly ranging from 10 to 100 ms and from −200 to 200 Hz, respectively.

Figure 5 displays the magnitude and phase evolution of seven randomly selected dictionary entries. The magnitude signal follows the exponential decay, while phase wrappings occur with large field inhomogeneity and long echo train readout. More importantly, the SVD analysis of the signal dictionary shows that at least 26 principal components are required to represent the complex signal behaviour.

### (b) Frequency-modulated SSFP

Conventional balanced steady-state free precession (bSSFP) sequences exhibit a high signal-to-noise ratio (SNR) but suffer from possible signal voids in regions with certain off-resonance distributions. These voids or banding artefacts can be removed when multiple images are acquired with different transmitter phase cycles. Foxall and co-workers demonstrated that bSSFP sequences are tolerant to small but continuous changes in transmitter frequency [53]. In [49], we exploited this method to develop a time-efficient alternative to phase-cycled bSSFP that waives intermediate preparation phases in phase-cycled bSSFP to establish different steady-states. Image reconstruction is performed in the low-frequency Fourier subspace and yields signal intensity and contrast comparable to on-resonant bSSFP.

**Figure 8.**
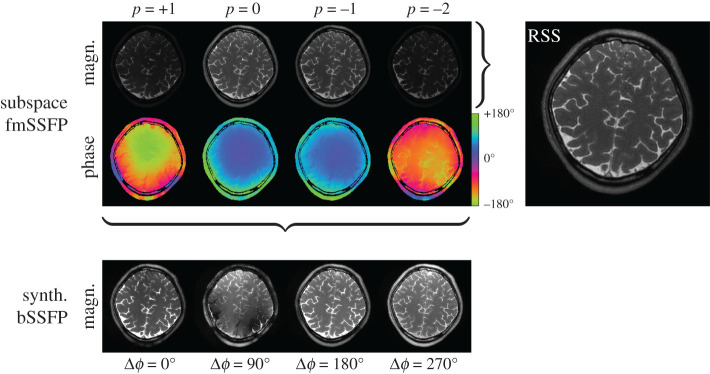
Reconstructed subspace coefficients maps (top) along with its root-sum-squares composite image for a individual slice within the acquired 3D volume. Synthesized bSSFP images are computed from these coefficient maps for different virtual frequency offsets (bottom). (Online version in colour.)

To this end, a frequency-modulated SSFP (fmSSFP) pulse sequence [53] was combined with 3D stack-of-stars data acquisition such that a single full sweep through the spectral response profile was obtained. Aligned partitions allowed us to decouple the reconstruction problem into individual slices by a 1D inverse Fourier transform. After coil sensitivity estimation [54], image reconstruction was performed by solving a linear subspace-constrained reconstruction problem using a local low rank regularization. As a subspace basis, the four lowest order Fourier modes were chosen. Figure 8 shows the reconstructed complex-valued coefficient maps from which a composite image can be computed in a root-sum-squares manner (top). Additionally, synthesized bSSFP images are computed for four virtual frequency offsets (bottom) in which the distribution of signal voids is given by the phase distribution of the subspace coefficients. These synthesized bSSFP images correspond to conventional bSSFP images acquired with four different phase cycles.

## 5. Discussion

In the past decades, various techniques were developed to accelerate quantitative MRI. One very general way is to exploit complementary information from spatially distinct receiver coils, called parallel imaging (PI) [55–57]. Others make use of the fact that MR images are usually sparse in a certain transform domain and combined with incoherent sampling and nonlinear image reconstruction algorithms it is called compressed sensing (CS) [58]. Exploiting this prior knowledge about a compressible image, CS can recover MR images from highly undersampled data [59,60]. Other approaches combine PI and CS with efficient non-Cartesian sampling schemes [60].

When it comes to parameter mapping, besides the already mentioned sparsity constraints, low-rank constraints or joint sparsity can also be exploited along the parameter dimension to accelerate the acquisition [14,16,61–63].

Generally speaking, the methods above usually consist of two steps: first reconstruction of contrast-weighted images from undersampled datasets and second, the subsequent voxel-by-voxel fitting/matching. By contrast, model-based reconstructions integrate the underlying MR physics into the forward model, enabling estimation of MR physical images (parameter maps) directly from the undersampled *k*-space, bypassing the intermediate steps of image reconstruction and pixel-wise fitting/matching completely. This has the advantage of only reconstructing the desired parameter maps instead of a set of contrast-weighted images, i.e. reducing the number of unknowns tremendously. Another advantage is that parameter estimation using L2 norm in the data fidelity is optimal (assuming white Gaussian noise), while fitting reconstructed magnitude images may introduce a noise-dependent bias. Special sampling strategies are required for model-based reconstruction to achieve good reconstructions. Sampling schemes used include CAIPIRINHA [64] and golden-ratio radial acquisition [65].

In contrast to nonlinear model-based reconstruction methods which use a minimal number of physical parameters to describe the MR signal precisely, linear subspace methods approximate the MR signal using a certain number of principal coefficients. As discussed above, this is computationally much more efficient and avoids partial volume effects. Subspace methods were also successfully used for multi-parametric imaging, for example using pseudo steady-state free precession (pSSFP) [48] or echo planar time-resolved imaging where it is combined with nonlinear iterative phase estimation [52]. Subspace methods have to balance two additional error terms coming from 1) the approximation error when the subspace size is too small and 2) noise amplification when the subspace size is large (more unknowns). To minimize these additional errors, the optimal subspace size has to be selected. While noise can be predicted based on the size of the subspace, the approximation error is more difficult to control and may require systematic studies that include comparisons to a ground truth.

Model-based reconstructions are, in general, memory demanding and time consuming as all the data has to be held in the memory simultaneously during iterations. However, modern computational devices such as GPUs have enabled faster reconstructions. For example, the computation time for model-based *T*_1_ reconstruction presented here has been reduced from around 4 h in CPU (40-core 2.3 GHz Intel Xeon E5–2650 server with a RAM size of 512 GB) to 6 min using GPUs (Tesla V100 SXM2, NVIDIA, Santa Clara, CA). Other smart computational strategies [25,46,66] may also be employed to reduce the memory and computational time. The other limitation might be that model-based reconstructions are sensitive to model mismatch, e.g. bi-exponential processes, slow exchange regime. One way to overcome such limitations is to explicitly model these effects and include them into the model-based reconstruction.

Validation or the assessment of errors is an important part of developing nonlinear model-based reconstruction methods. To this end, several strategies should be applied. First, numerical simulations with analytical k-space models can ensure general convergence and robustness to noise as noise levels can be freely chosen and noise-free ground truth is available. Second, *in vitro* or phantom studies covering a certain range of parameters of interest should be performed as effects such as intra-voxel dephasing, imperfect RF excitation, and shimming are hard to simulate and the effects of such model errors are hard to predict. Several hardware phantoms are commercially available, well characterized and widely used [67]. Last, *in vivo* measurements should always be evaluated against established methods or fully sampled datasets if possible.

Tremendous progress in the fields of machine learning/deep learning has sparked a huge interest in applying these methods to different MRI applications including image reconstruction [68,69]. However, so far only a few applications exist that target accelerated parameter mapping directly [70–73]. While these are promising developments, there are also still unsolved questions regarding the stability of machine learning methods [74] and the risk of introducing image features that look real but are not present in the data (hallucinations) [75].

Magnetic resonance fingerprinting (MRF) [76] is an alternative technique to perform time-efficient multi-parametric mapping leveraging high undersampling factors. In its original formulation, parameter maps are reconstructed in a two-step procedure. First, time series are generated by an inverse NUFFT operation agnostic to any physical signal model. Second, parameter maps are generated by pixel-wise matching of the obtained time series with a precomputed dictionary consisting of simulated signal prototypes. The proposed decoupling into a linear reconstruction of time series and a nonlinear fitting problem solved by exhaustive search results in comparatively short reconstruction times and does not require analytical signal models. These two advantages have rendered MRF a very popular approach in recent years. This two-step procedure, however, comes at a cost. The initial model-agnostic gridding operation results in heavily aliased signal time courses. Aliasing can be removed only partially by pixel-wise matching, as no information on the sampling pattern is available in that step, and might deteriorate or bias the obtained parameter maps. Recent studies have tried to overcome this inherent drawback of the two-step method by iterating between time and parameter domain [77] or by formulating the reconstruction as a nonlinear problem that integrates the physical signal model and additional image priors [7] similar to the discussed model-based approaches. Techniques combining iterative reconstructions and grid searches on dictionaries were also developed [78]. For a recent review that discusses the basic concept of MRF in the context of other quantitative methods see [79].

## 6. Conclusion

By formulating image reconstruction as an inverse problem, model-based reconstruction techniques can estimate quantitative maps of the underlying physical parameters directly from the acquired k-space signals without intermediate image reconstruction. While this is computationally demanding, it enables very efficient quantitative MRI.

## Data accessibility

All model-based reconstructions were performed with the BART toolbox. Scripts to reproduce the examples shown in this work are available at https://github.com/mrirecon/physics-recon. Data are available at doi: 10.5281/zenodo.4381986.
